# Letheon

**Published:** 1847-04

**Authors:** 


*Letheon.*—The somnific vapor has been introduced into France and England, and appears to be the the engrossing medical topic in both countries at the present time. Mr. Liston, of London, has performed several capital operations upon patients rendered insensible by it ; and Velpeau, and others, at Paris, have tried it with the same success. Foreign Periodicals reprobate, as do those generally of this country, the attempt to secure for it a patented restriction. At Boston the credit of the discovery is being warmly contested by Drs. Jackson and Morton. Each of these gentlemen claims priority, and as the contest appears now to rest upon the question of veracity, as regards both the parties, we wait for farther evidence before venturing to form an opinion on the subject. One false step is sure to lead to a succession of deviations from the right path. We cannot forbear the reflection that had not a mercenary, unprofessional spirit been allowed to have had supremacy in the first instance, the occasion for the present contention would probably not have existed.

